# The Wedelolactone Derivative Inhibits Estrogen Receptor-Mediated Breast, Endometrial, and Ovarian Cancer Cells Growth

**DOI:** 10.1155/2014/713263

**Published:** 2014-08-13

**Authors:** Defeng Xu, Tzu-Hua Lin, Chiuan-Ren Yeh, Max A. Cheng, Lu-Min Chen, Chawnshang Chang, Shuyuan Yeh

**Affiliations:** ^1^School of Pharmaceutical and Life Sciences, Changzhou University, Changzhou, Jiangsu 213164, China; ^2^George H. Whipple Laboratory for Cancer Research, Departments of Urology and Pathology, University of Rochester Medical Center, 601 Elmwood Avenue, Rochester, NY 14642, USA; ^3^Sex Hormone Research Center, China Medical University/Hospital, Taichung 404, Taiwan

## Abstract

Estrogen and estrogen receptor (ER)-mediated signaling pathways play important roles in the etiology and progression of human breast, endometrial, and ovarian cancers. Attenuating ER activities by natural products and their derivatives is a relatively practical strategy to control and reduce breast, endometrial, and ovarian cancer risk. Here, we found 3-butoxy-1,8,9-trihydroxy-6H-benzofuro[3,2-c]benzopyran-6-one (BTB), a new derivative of wedelolactone, could effectively inhibit the 17-estradiol (E2)-induced ER transactivation and suppress the growth of breast cancer as well as endometrial and ovarian cancer cells. Our results indicate that 2.5 *μ*M BTB effectively suppresses ER-positive, but not ER-negative, breast, endometrial, and ovarian cancer cells. Furthermore, our data indicate that BTB can modulate ER transactivation and suppress the expression of E2-mediated ER target genes (Cyclin D1, E2F1, and TERT) in the ER-positive MCF-7, Ishikawa, and SKOV-3 cells. Importantly, this BTB mediated inhibition of ER activity is selective since BTB does not suppress the activities of other nuclear receptors, including glucocorticoid receptor and progesterone receptor, suggesting that BTB functions as a selective ER signaling inhibitor with the potential to treat breast, endometrial, and ovarian cancers.

## 1. Introduction

Breast, endometrial, and ovarian cancers are the three most common cancers among women in the United States and are the major causes of cancer related deaths in females. Breast, endometrial, and ovarian cancers are associated with excessive estrogen exposure. Based on hormonal responsiveness, the development of effective prevention strategies for breast, endometrial, and ovarian cancers is a paramount opportunity in the care of women at risk for those malignancies [1].

Estrogens are pleiotropic hormones that regulate the growth and differentiation of many diverse tissues. The action of estrogens is mediated through the estrogen receptor (ER), which belongs to the nuclear hormone receptor superfamily of ligand-activated transcription factors [2]. ER has two major subtypes, ER*α* and ER*β*, each of which predominates in specific tissues and organs. Estrogens play crucial roles in many types of gynecological malignancies by modulating or substituting for ER-dependent activities [3]. It is now well established that estrogens play a fundamental role in promoting the growth and progression of ER-related cancer cells. This understanding and control of the ER pathways have led to the development of various endocrine therapies by blocking estrogen-mediated tumor growth and progression.

The proliferation of estrogen-dependent cancers is reduced by antiestrogen treatment in both dose- and time-dependent manners [4]. ICI 182,780 (ICI), a pure antiestrogen, is a steroidal molecule that can inhibit E2-mediated activity and induce apoptosis in estrogen-dependent cancer cells.

**Figure 1:**
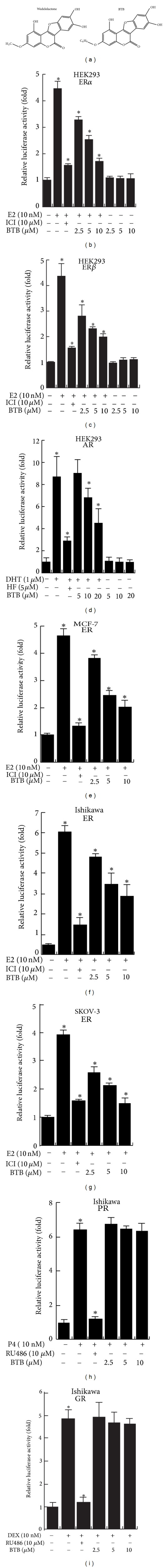
BTB selectively inhibits E2-mediated ER transactivation, moderately inhibits DHT-mediated AR transactivation, and fails to inhibit the PR and GR activity. (a) Chemical structures of wedelolactone and BTB. (b) and (c) BTB at 2.5, 5.0, and 10 *μ*M concentrations can effectively inhibit the estrogen-induced ER*α* or ER*β* transcriptional activity in HEK293 cells. (d) BTB moderately inhibits the androgen-induced AR transcriptional activity in HEK 293 cells. BTB at 5.0 *μ*M concentration could not inhibit the AR transactivation, and 10 *μ*M of BTB treatment started to show about 15–20% inhibition of the AR transactivation. (e)–(g) Inhibition of BTB on the E2-induced ER transcriptional activity in MCF-7, Ishikawa, and SKOV-3 cells. (h) and (i) Noeffects of BTB on the transcriptional activities of P4-induced PR and Dex-induced GR in Ishikawa cells. MMTV-Luc was used to determine the AR, PR, and GR mediated transactivation. ERE-Luc was used to assay ER*α* and ER*β* mediated transactivation activities. Data represent mean ± SD collected from three independent experiments with duplication in each experiment.

Traditional herb plants have a great importance due to their potential uses to treat diseases.* Wedelia chinensis *(Family: Asteraceae) has been used as a traditional Chinese medicine to treat septic shock, liver diseases, and viral infections [5]. This plant is a source of several secondary metabolites, such as flavonoids, phytosterols, and coumestans [6]. Coumestans represent an important class of natural oxygenated aromatic products, including wedelolactone. Wedelolactone (Figure 1(a)) is a naturally occurring furanocoumarin, which is an inhibitor of the IKK II [7], a kinase critical for the activation of NF-*κ*B by mediating phosphorylation and degradation of I*κ*B*α*. It also has many different bioactivities including antihepatotoxic, antihypertensive, antitumor, and antiphospholipase A2 properties, as well as being used as an antidote against snake venoms [8–11]. In addition to controlling NF-*κ*B activity, wedelolactone is also known to possess estrogenic activity as it belongs to the coumestans family [12]. Recently, it was reported that wedelolactone inhibits growth of prostate and pituitary cancer cells [13–15].

To enhance and improve the anticancer activity of wedelolactone, we modified the wedelolactone chemical structure and tested the bioactivities of ten new derivatives. Among those new derivatives, we found that 3-butoxy-1,8,9-trihydroxy-6H-benzofuro[3,2-c]-benzopyran-6-one (BTB) had antiestrogen activities. Its chemical structure is shown in Figure 1(a). This is the first study to discover and characterize the ability of BTB in regulating ER transactivation. We also analyzed BTB's effects on mRNA expressions of ER target genes in different ER-positive cancer cells. It has been reported that estrogen/ER can activate the c-Myc and Cyclin D1 expression. Our data showed that the c-Myc and Cyclin D1 protein expression is regulated by estrogen/ER*α*, and the 17*β*-estradiol (E2)/ER-induced c-Myc/Cyclin D1 expression can be blocked by BTB treatment. We further investigated the the effects of BTB on cell growth in the estrogen-stimulatory environment using ER-positive versus ER-negative breast, endometrial, and ovarian cancer cell lines. Together, our data showed BTB could inhibit ER activity via decreasing the ER protein. BTB is a selective ER signaling inhibitor with the potential as therapeutic treatment for breast, endometrial, and ovarian cancers.

## 2. Materials and Methods

### 2.1. Chemicals and Reagents

At Shanghai Jiaotong University we modified wedelolactone and prepared ten new derivatives. We used the Sonogashira coupling reaction of 2-bromo-4,5-dibenzyloxyaniline with substituted phenylacetylene, a diazotization hydrolysis reaction, a ring closure, and deprotection reaction to obtain new derivatives. We screened their capability to inhibit ER and AR activities and found that the BTB had better antiestrogen activities. Commercial compounds and reagents including E2, ICI, dexamethasone (DEX), Mifepristone (RU-486), progesterone (P4), ethanol (EtOH), ethyl acetate (EtOAc), methanol, and chloroform (CHCl_3_) were purchased from Sigma (St. Louis, MO, USA). All other chemicals and solvents used in this study were of reagent or high performance liquid chromatography (HPLC) grade.

### 2.2. Plasmids

The plasmids used were pcDNA3.1-ER*α*, mouse mammary tumor virus (MMTV)-luciferase (Luc) reporter plasmid; plasmids pSG5-androgen receptor (pSG5-AR), pSG5 progesterone receptor (pSG5-PR), pSG5 glucocorticoid receptor (pSG5-GR), pWPI-Flag-ER*β*, pGL3(ERE)_3_-Luc, and pRL-TK were constructed as described previously [16–21].

### 2.3. Western Blot Analysis

50 *μ*g of protein/lane was used for the analysis. The blots were probed with primary anti-ER*α* (Novocastra, 6F11), anti-c-Myc (cell signaling, #9402), anti-Cyclin D1 (#2922), anti-Stat3 (#9132), anti-pStat3 (#9145), anti-Akt (#9272), and anti-pAkt(S-473) (#9271) antibodies with dilutions of 1 : 500 to 1 : 1,000 and incubated at room temperature for 2 hrs. The secondary antibodies, horseradish peroxidase-conjugated anti-rabbit and anti-mouse IgG, were used at room temperature for 1 hr. Immunoblot analysis was performed using enhanced chemiluminescence Western blotting detection reagents (Amersham Biosciences).

### 2.4. Cytotoxicity Assay* In Vitro*

Cytotoxicity assay was performed according to the protocol described in our previous publication [22]. To determine the IC_50_ value, 6.0 × 10^4^ MDA-MB-231, Ishikawa, SKOV-3, HEC-1-A, and OVCA429 cells were plated in triplicate in 5% CS-FBS DMEM in 24-well culture plates. The cells were incubated with serial concentrations of BTB for 2 days and cell viability was determined by MTT assay. BTB + medium only (no cells) were included as a baseline reading to subtract the reading background. BTB treated cells were compared to DMSO treated cells. IC_50_ value was analyzed with the program CompuSyn (Developer). MCF-7 cells were seeded using RPMI-1640 with 5% CS-FBS medium. The IC_50_ of BTB in MCF-7 cells was tested with the same methods.

### 2.5. Statistics

Data are presented as the means ± SDs for the indicated number of separate experiments. The statistical significance of differences between two groups of data was analyzed by paired* t*-test and *P* values <0.05 were considered significant.

*Other methods* including cell culture, luciferase assay, RNA extraction, reverse transcription, real-time PCR, and cell growth assay* in vitro *were described in the Supplementary Information (Doc. S1) available online at http://dx.doi.org/10.1155/2014/713263.

## 3. Results

### 3.1. BTB Selectively Inhibits the E2-Mediated ER Transactivation, Has a Less Inhibition Efficacy toward AR, and Fails to Inhibit the PR or the GR Mediated Transactivation

Nuclear receptors are ligand-dependent transcription factors that control a variety of essential physiologic and developmental processes as well as disease progression in humans [23]. For example, estrogen-ER signals were found to be crucial for breast, endometrial, and ovarian cancer development. As BTB belongs to the furanocoumarin family/group compound, which could possess the agonist or antagonist activity of ER, we first investigated the ability of BTB to regulate ER transactivation activity in the HEK 293 cells. The relative luciferase activity was determined within cells transiently transfected with ERs (ER*α* or ER*β*) and an ERE-luciferase reporter construct, pGL3(ERE)_3_-Luc. ER's transactivation activity was induced by E2 but blocked by the antiestrogen, ICI. Furthermore, our data showed that BTB was able to suppress E2-induced ER transactivation in a dose-dependent manner (Figures 1(b)-1(c)). In addition, we found 2.5 and 5.0 *μ*M BTB treatments could effectively inhibit the ER*α* and ER*β* transactivation but did not inhibit the AR transactivation. However, only when the concentration of BTB reached 10 *μ*M did it start to show about 15–20% inhibition of the AR transactivation (Figure 1(d)).

To further confirm the ability of BTB to control ER transactivation activity, we used the ER-positive breast, endometrial, and ovarian cancer cells, MCF-7, Ishikawa, and SKOV-3, respectively, transfected with ERE-Luc. ER transactivation activities were induced by 10 nM E2 and could be blocked by ICI. Our data showed that BTB at the concentration of 2.5, 5.0, and 10 *μ*M could effectively suppress E2-ER transactivation in the ER-positive MCF-7 breast cancer cells, Ishikawa endometrial cancer cells, and SKOV-3 ovarian cancer cells (Figures 1(e)–1(g)).

We further evaluated the effect of BTB on P4-induced PR transcriptional activity in Ishikawa cells transfected with PR and a MMTV-luciferase reporter. PR transactivation activity was induced by 10 nM P4 and blocked by 10 *μ*M RU486. However, BTB had no inhibitory effect on P4-mediated PR transactivation (Figure 1(h)). In comparison, we tested whether BTB can inhibit the GR mediated transactivation, and as shown in Figure 1(i), the antagonist RU486 can, but BTB cannot, inhibit the Dex-GR activities. Together, 2.5 or 5 *μ*M BTB treatment selectively inhibits ER activity but not AR, PR, or GR transactivation. 10 *μ*M BTB starts to show a marginal 15%–20% inhibitory effect on AR and still has no effect on PR and GR transactivations.

### 3.2. BTB Inhibits the E2-Induced Growth in ER-Positive, but Not in ER-Negative, Breast, Endometrial, and Ovarian Cancer Cells

**Figure 2:**
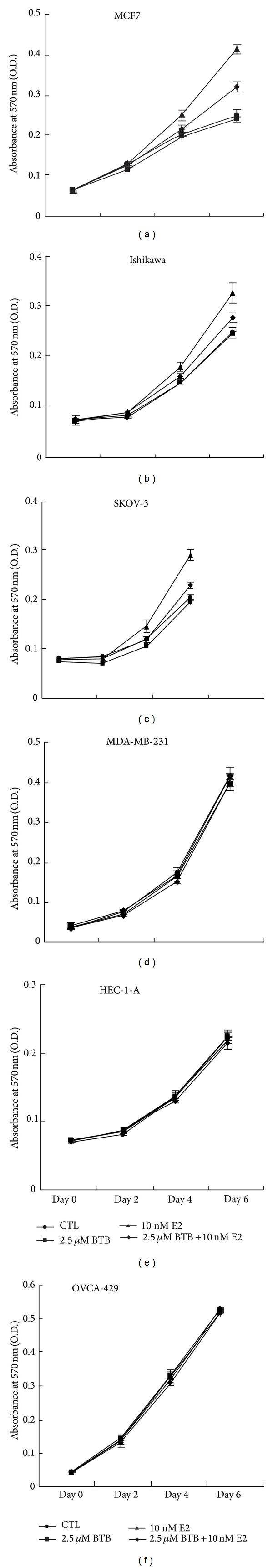
Differential growth inhibition effects of BTB on ER*α*-positive versus ER*α*-negative breast, endometrial, and ovary cancer cell lines. Cells were treated with 2.5 *μ*M BTB or mock EtOH in the absence or presence of 10 nM E2. Phenol red free DMEM medium with indicated treatment was refreshed every 2 days for a total of 6 days. (a)–(c) BTB Inhibits the E2-induced growth of ER-positive breast cancer MCF-7, endometrial cancer Ishikawa, and ovarian cancer SKOV-3 cells. (d)–(f) BTB has no effect on the growth of ER-negative breast cancer MDA-MB-231, endometrial cancer HEC-1-A, and ovarian cancer OVCA429 cells. Data represent mean ± SD collected from three independent experiments.

As BTB could selectively inhibit the ER-mediated transactivation, we then investigated the effects of BTB on the inhibition of E2-induced cell proliferation in ER-positive MCF7, Ishikawa, and SKOV-3 cancer cells and compared this with the ER-negative MDA-MB-231, HEC-1-A, and OVCA429 cancer cells. BTB reduced E2-induced cell growth in MCF-7, Ishikawa, and SKOV-3 cells by 50%, 55%, and 62%, respectively (Figures 2(a)–2(c)). As shown in Figure 1, 2.5 *μ*M BTB treatment could not completely block E2-induced ER transactivation on ERE-luciferase; we did not expect to see 100% completion of E2 stimulated growth. On the other hand, BTB failed to inhibit the growth of those three ER-negative cancer cell lines in the presence or in the absence of E2 (Figures 2(d) and 2(f)).

**Figure 3:**
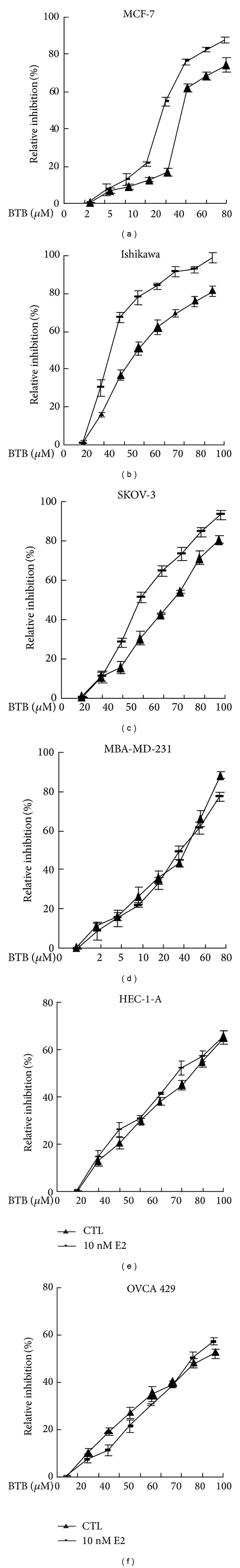
IC_50_ of BTB in different breast, endometrial, and ovary cancer cell lines. We determine the cell half-inhibition (IC_50_) of BTB in MCF-7, Ishikawa, SKOV-3, MDA-MB-231, HEC-1-A, and OVCA429 cells. Cells were seeded on 24-well plates in medium with 10% FBS for 24 hr. Medium was then refreshed to phenol red free DMEM medium with 5% CS-FBS for another 24 hr, and cells were treated with serial concentrations of BTB with or without 10 nM E2 for 2 days. Cells growth and IC_50_ value were determined by MTT assay and confirmed by direct cell number count. The concentration of BTB treatment that leads to 50% inhibition of cell growth is determined as IC_50_. Data represent mean ± SD collected from three independent experiments.

The half maximal inhibitory concentration (IC_50_) can be determined by constructing a dose-response curve and examining the effect of different concentrations of antagonists on reversing the agonist activity [18, 24]. The IC_50_ concentrations for BTB on the breast, endometrial, and ovarian cancer cell lines were evaluated by half-inhibition of cell growth after 48 hr of BTB treatments in the presence or absence of 10 nM E2. In the presence of 10 nM E2, the IC_50_ concentrations for BTB in MCF-7, Ishikawa, SKOV-3, MDA-MB-231, HEC-1-A, and OVCA429 were 18.3 ± 2.0 *μ*M, 32.2 ± 2.0 *μ*M, 41.9 ± 2.5 *μ*M, 42.5 ± 3.5 *μ*M, 80.4 ± 4.5, and 77.5 ± 4.5 *μ*M, respectively (Figure 3). In the absence of E2, the IC_50_ concentrations for BTB in those cell lines were 29.6 ± 2.0 *μ*M, 63.2 ± 3.5 *μ*M, 79.6 ± 4.0 *μ*M, 43.8 ± 2.5 *μ*M, 84.3 ± 4.5 *μ*M, and 80.3 ± 4.5 *μ*M, respectively. As shown in Figure 1, 2.5 uM BTB treatment could not completely block E2-induced ER transactivation on ERE-luciferase. Therefore, we did not expect to see that BTB could 100% inhibit E2 stimulated growth. Together, we used multiple assays and reproducibly found that BTB could effectively inhibit the ER-positive MCF-7, Ishikawa, and SKOV-3 cell growth. Therefore, we determined the mechanisms by which BTB controls ER activity and cancer cell growth in ER-positive MCF-7, Ishikawa, and SKOV-3 cells.

### 3.3. BTB Inhibits the E2-Induced ER Target Gene Expression in MCF-7, Ishikawa, and SKOV-3 Cells

As shown in Figure 1, we observed that 2.5 *μ*M BTB could effectively inhibit ER/ERE-Luc activity. Although our results show BTB can inhibit both ER*α* and ER*β*, ER*α* seems to play a more dominant role in those 3 female cancers. Earlier reports showed that ER*α*, but not ER*β*, is overexpressed in breast, endometrial, and ovarian cancers [25–27].

**Figure 4:**
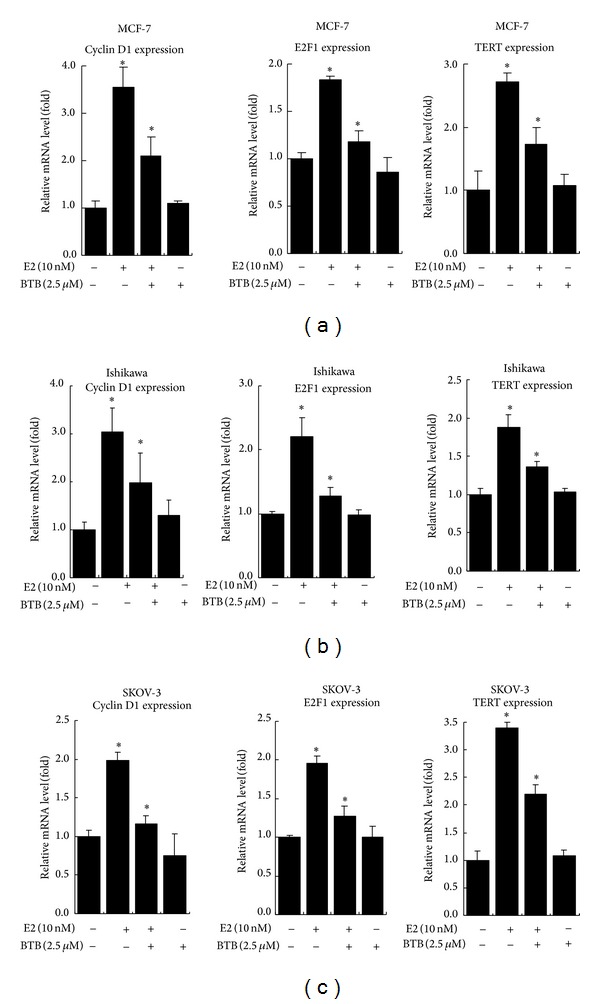
BTB inhibits the mRNA expression of ER target genes in MCF-7, Ishikawa, and SKOV-3 cells. Cells were treated with 2.5 *μ*M BTB or EtOH control in the absence or presence of 10 nM E2 for 24 hrs. We used real-time RT-PCR to analyze the mRNA expressions of ER target genes, Cyclin D1, E2F1, and TERT, in MCF-7, Ishikawa, and SKOV-3 cells. The mRNA levels of these genes in each treatment group were displayed as fold changes compared to the untreated group. Data are shown as the mean ± SD of three independent experiments with three replicates in each experiment.

Therefore, we focused on the ER*α* in the following studies. To further illustrate the ability of BTB to regulate the ER downstream pathway, we assayed ER target genes in the ER-positive cells, MCF-7, Ishikawa, and SKOV-3 treated with 25 *μ*M BTB treatment. Our data show that the mRNA expression of ER target genes, Cyclin D1, E2F1, and TERT [28–30], was induced by 10 nM E2, and the treatment with 2.5 *μ*M BTB could effectively suppress the E2-induced ER target genes expression in MCF-7, Ishikawa, and SKOV-3 cells (Figure 4).

### 3.4. BTB Inhibits the E2-Induced ER*α*, c-Myc, and Cyclin D1 Protein Levels in MCF-7, Ishikawa, and SKOV-3 Cells

**Figure 5:**
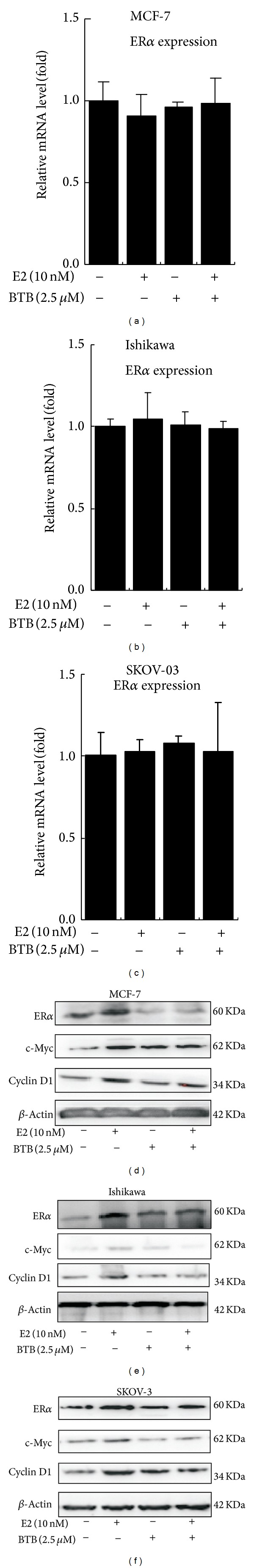
BTB reduces ER*α*, c-Myc, and Cyclin D1 protein expression levels in MCF-7, Ishikawa, and SKOV-3 cells but has no effect on ER*α* gene expression at mRNA level. Western blot analyses of ER*α*, c-Myc, and Cyclin D1 levels in control and 2.5 uM BTB treated MCF-7, Ishikawa, and SKOV-3 cells in the absence or presence of 10 nM E2. 50 *μ*g of total protein from cells was applied onto a 10% sodium dodecyl sulfate-polyacrylamide gel and subjected to electrophoresis followed by Western blot using anti-ER*α*, anti-c-Myc, or anti-Cyclin D1 antibodies. Representative graphs were shown from consistent results collected from three independent experiments.

Our data showed that BTB inhibited the ER mediated activity and target gene expression, yet the mechanism remains unclear. At the same time, using RT-PCR, we observed that the treatment with BTB did not change the ER*α* mRNA levels in MCF-7, Ishikawa, and SKOV-3 cells (Figures 5(a)–5(c)). Our data showed that BTB treatment significantly inhibited the protein levels of ER*α* and E2-induced target genes (Figures 5(d)–5(f)).

We also determined the effect of BTB on E2-induced protein levels of c-Myc and Cyclin D1 in MCF-7, Ishikawa, and SKOV-3 cells. Our data showed that the protein levels of c-Myc and Cyclin D1 were induced by E2, and BTB significantly inhibited the c-Myc and Cyclin D1 protein expressions (Figures 5(d)–5(f)).

### 3.5. BTB Fails to Affect the Expression Levels and the Phosphorylation Status of Akt and STAT3 in ER-Positive Cancer Cells

In addition to E2/ER*α*, it has been documented that Akt and STAT3 are important cancer survival pathways in those cancer cells [31, 32]. Our data showed that BTB could effectively inhibit the growth of MCF-7, Ishikawa, and SKOV-3. Other than ER*α*, it is of great interest to test whether BTB can affect the activity of Akt or STAT3. Cancer cells were seeded in phenol red free DMEM media with 10% CS-FBS for 48 hrs and then treated with BTB or EtOH in the presence or absence of 10 nM E2 for 24 hrs. The cells were harvested and lysates were subjected to electrophoresis and Western blotting analyses.

**Figure 6:**
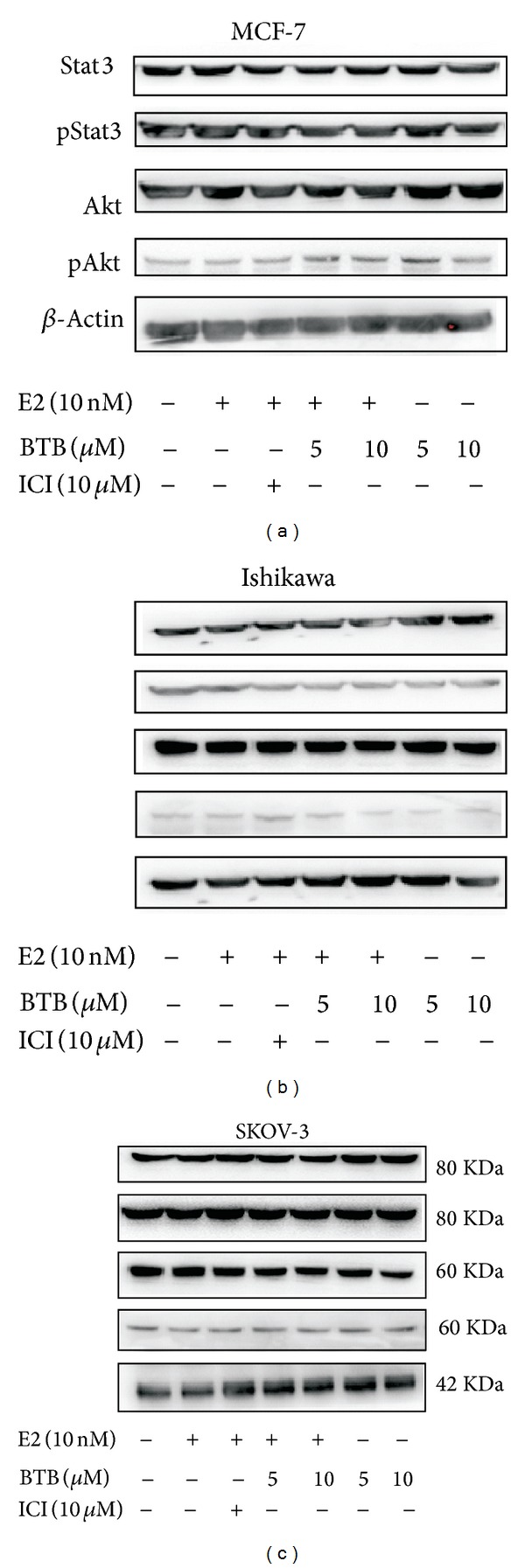
BTB does not affect the phosphorylation of Akt and Stat3 expression in MCF-7, Ishikawa, and SKOV-3 cells. Western blot analyses of Akt, p-Akt, Stat3, and p-Stat3 levels in control and 5 and 10 *μ*M BTB treated MCF-7, Ishikawa, and SKOV-3 cells in the absence or presence of 10 nM E2. 50 *μ*g of protein lysate of each sample was applied onto a 10% sodium dodecyl sulfate-polyacrylamide gel and subjected to electrophoresis followed by Western blot using anti-AKT, anti-p-AKT (s473), anti-Stat3, and anti-p-Stat3 antibodies. Representative graphs were shown from consistent results collected from three independent experiments.

Our data showed that the BTB mediated growth suppression did not affect the expression level of the phosphorylation of Akt (s473) and STAT3 in ER-positive cancer cells (Figures 6(a)–6(c)) and demonstrated the specificity of BTB effects on ER signaling pathway to modulate growth and survival of 3 major types of female cancers.

## 4. Discussion

Hormonal therapy targeting ER signaling is one of the most effective treatments for steroid hormone related cancer. In the current study, we showed that the new derivative compound BTB, which was modified from wedelolactone, has the potential in breast cancer and gynecological cancer therapy by inhibition of ER signaling.

The chemical structure of wedelolactone designates it as part of the coumestans family, which is known to possess estrogenic activity [12]. Recently, it was shown that wedelolactone is able to suppress AR activity and inhibit cell growth in AR positive prostate cancer cells [13–15]. Interestingly, the new derivative BTB at 2.5 or 5.0 *μ*M concentrations can efficiently inhibit both ER*α* and ER*β* transcriptional activity but only inhibit AR activity at a higher dose. Meanwhile, other steroid receptor signaling pathways, including PR and GR, are not affected even at the higher doses, indicating that BTB selectively inhibits ER signaling. In addition, 2.5 or 5.0 *μ*M of BTB treatments for 24 or 48 hrs could not induce apoptotic signals in those 3 cancer cell lines (data not shown). It was reported that several signaling pathways, like phosphorylation of Akt or STAT3, may contribute to the cancer cells growth and progression [33–37]. However, the BTB mediated growth suppression did not affect those signal pathways and was only effective in ER-positive cancer cells, again demonstrating the specificity of BTB effect on the ER signaling pathway.

The estrogen antagonists can inhibit the transcriptional activity of ER, but some of the estrogen-dependent cancer cells can escape this competitive inhibition and activate the ER signaling pathway by growth factors or coactivators, suggesting that the direct targeting on ER protein could be an important therapeutic strategy in those advanced cancer cells [38, 39]. Our data showed that BTB could decrease the E2-induced ER*α* protein level but not the mRNA level, indicating that the inhibition effect may function through translational repression or protein degradation. It would be interesting to study how BTB reduces ER protein level and what is the potential implication in cancer therapy.

In the estrogen-related cancer cells, many of the ER*α* downstream target genes were reported to play important roles in cell growth and survival, which may be linked to tumor progression. In those ER-related cancer cells, E2-activated ER*α* modulates the expression of key cell cycle regulatory genes, including Cyclin D1 [30, 40] and the transcription factor E2F1 [28]. These factors play important roles in cell cycle progression, directing the phosphorylation and inactivation of the retinoblastoma protein and mediating the expression of genes involved in DNA replication and S phase entry [41].

Human telomerase reverse transcriptase (hTERT) is a catalytic subunit of telomerase. Some studies have found that hTERT is expressed in most malignant tumors, but not in normal somatic cells, and that its expression is closely associated with telomerase activity [29]. E2 activates hTERT transcription through the direct interaction of ligand-activated ER with the ERE sequence located at 72677 in the hTERT 5′ regulatory region in endometrial cancer cells [42], implying the existence of hormone-dependent control mechanisms of telomerase activity. The overexpression or amplification of c-Myc was observed in many types of cancers [43]. It was also proven that c-Myc could play important roles in cancer cell growth, apoptosis, metabolism, and cell differentiation [44]. Since it was demonstrated that E2 treatment could enhance the c-Myc expression in ovarian cancer cells [45], inhibition of c-Myc expression through blocking estrogen signaling may also contribute to the suppression of cancer progression.

In brief, the current data indicated that our new wedelolactone derivative BTB can specifically inhibit the ER signaling and block the E2 stimulated cell proliferation in the estrogen-related cancers. Decreased ER*α* protein was observed, and the ER*α* downstream target protein was also reduced in BTB treated cells. The potential clinical application of BTB use in the treatment of breast cancer and possibly in other gynecological cancers will be interesting for the further study.

## Supplementary Material

The supplementary document includes detailed methods for I. Cell Culture, II. In Vitro Cell Growth Assay, III. Luciferase Assay, and IV. RNA Extraction, Reverse transcription, and Real-time PCR.

## Abbreviations

AR: Androgen receptor
BTB: 3-Butoxy-1,8,9-trihydroxy-6H-benzofuro[3,2-c] benzopyran-6-one
DEX: Dexamethasone
ER: Estrogen receptor
ERE: Estrogen-responsive element
E2: 17*β*-Estradiol
ICI: ICI 182,780
GR: Glucocorticoid receptor
Luc: Luciferase
P4: Progesterone
PR: Progesterone receptor
hTERT: Human telomerase reverse transcriptase.
