# Supplementary material for: The Wedelolactone Derivative Inhibits Estrogen Receptor-Mediated Breast, Endometrial, and Ovarian Cancer Cells Growth
Source: Biomed Res Int. 2014 Aug 13;2014:713263. doi: 10.1155/2014/713263 (PMC4157183; doi:10.1155/2014/713263)
Supplement: Supplementary file 1 — The supplementary document includes detailed methods for I. Cell Culture, II. In Vitro Cell Growth Assay, III. Luciferase Assay, and IV. RNA Extraction, Reverse transcription, and Real-time PCR. [file 713263.f1.zip › 713263.f1/SupplementaryDocument.docx]

# *The supplementary document includes detailed methods for I. Cell Culture, II. In Vitro Cell Growth Assay, III. Luciferase Assay, and IV. RNA Extraction, Reverse transcription, and Real-time PCR.*

# *I. Cell Culture*

All cells were obtained from ATCC and cultured at 5% CO2 and 37℃. ER positive breast cancer MCF-7 cells were maintained in RPMI-1640 medium containing 10% fetal bovine serum (FBS). ER negative breast cancer MDA-MB-231 cells, originally derived from a pleural effusion, were maintained in DMEM medium containing 10% FBS. Ishikawa cells express both estrogen and progesterone receptors and were established from a well-differentiated adenocarcinoma of the endometrium. HEC-1-A cells are ER negative endometrial cancer cells, derived from adenocarcinoma of human endometrium. ERα positive SKOV-3 and ER-negative OVCA429 ovarian cancer cells were maintained in DMEM containing 10% FBS. HEK293 cells were generated by transformation of human embryonic kidney cells, and were maintained in DMEM with 10% FBS.

# *II. In Vitro Cell Growth Assay*

To determine cell growth, the MDA-MB-231, Ishikawa, HEC-1-A cells, and OVCA429 cells were plated at a density of 6×10^3^ cells per well in 24-well plates and allowed to attach overnight. After being incubated with 10% charcoal-stripped (CS)-FBS media for 24 hrs, these cells were treated with vehicle, 10 nM E2, 2.5 µM BTB, or 10 nM E2 + 2.5 µM BTB in medium with 2% CS-FBS and media with indicated treatments were refreshed every 48 hrs for a total of 6 days. Cell growth was determined by MTT assay (Sigma). Serum-free media containing 0.5 µg/ml MTT was added into each well. After 2 hrs incubation at 37℃, all crystals had solubilized and the optical density of the solution was determined spectrophotometrically at 570 nm.

The growth of SKOV3 cells is insensitive to E2 stimulation in DMEM medium, so they were grown in serum-free OPTI-MEMI to determine the E2 stimulated growth. The SKOV-3 cells were plated at a density of 3×10^4^ cells per well in 24-well plates overnight. These cells were then treated with vehicle, 10 nM E2, 2.5 µM BTB, or 10 nM E2 + 2.5 µM BTB in serum-free OPTI-MEM by refreshing culture media and treatment(s) every 2 days for a total of 6 days. MCF-7 cells were plated at 3×10^4^ cells/well on 24-well plates in RPMI-1640 with 10% CS-FBS for overnight. These cells were then treated with vehicle, 10 nM E2, 2.5 µM BTB, or 10 nM E2 + 2.5 µM BTB in RMPI-1640 by refreshing culture media and treatment(s) every 2 days for a total of 6 days. Cell growth/viability was determined by MTT assay.

# *III. Luciferase Assay*

# Transfections of reporter plasmid were performed using Lipofectamine 2000 (Invitrogen) according to the manufacturer’s protocol. HEK 293 cells, lacking functional ERα, ERβ, and AR were transfected with wild-type ERα, ERβ, or AR expression plasmid and reporter gene. Briefly, 2×10^4^ HEK 293 cells were plated on 24-well dishes with 10% CS-FBS DMEM media for 24 hrs, media was refreshed and cells were transfected with pcDNA3.1-ERα (or ERβ), pGL3 (ERE)_3_-Luc and pRL-TK-Luc for ERα (or ERβ) transactivation assay. Cells were transfected with pSG5-AR, MMTV-Luc, and pRL-TK-Luc to determine AR transactivation. After 24 hrs transfection, the media were refreshed to 10% CS-FBS media and treated with indicated concentrations of BTB or anti-estrogen (10 µM ICI 182,780) in the absence or presence of 10 nM E2 for 24 hrs. Cells were then harvested for luciferase assays.

ER**α** positive MCF-7, Ishikawa, or SKOV-3 cells were plated at a density of 5×10^4^ on 24-well dishes with 10% CS-FBS media for 24 hrs, media was refreshed and cells were then transfected with pGL3 (ERE)_3_-Luc and pRL-TK-Luc for 24 hrs. After transfection, the media was changed to 10% CS-FBS media and treated with indicated concentrations of BTB in the presence or absence of 10 nM E2 and/or 10 µM ICI for 24 hrs. For the GR and PR reporter activity assay, pSG5-PR, pSG5-GR, MMTV-Luc, and pRL-TK-Luc were transfected into Ishikawa cells. 10 nM progesterone, or DEX was added. To inhibit the progesterone-PR or DEX-GR activities, 10 µM RU486 was added. These cells were then harvested and assayed for luciferase activity using the Dual Luciferase Assay System. Data were expressed as relative luciferase activity normalized to the internal Renilla luciferase control.

# *IV. RNA Extraction, Reverse Transcription, and Real-time PCR*

Total RNA was extracted from breast, endometrial, or ovarian cancer cells using Trizol (Invitrogen). Reverse transcription was performed using the Superscript first-strand synthesis kit (Invitrogen). Quantitative real-time PCR analyses were performed on an ABI PRISM 7700 Sequence Detector System using the SYBR Green PCR Master Mix kit (Invitrogen) according to the manufacturer’s instructions. After an initial incubation at 50℃ for 2 min and 10 min at 95℃, amplification was performed for 40 cycles at 95℃ for 20 s, 65℃ for 20 s, and 72℃ for 30 s. Specific primer pairs were determined with the Primer-Express program (Applied Biosystems). The ERα primer pairs were 5'-GAC AGG GAG CTG GTT CAC ATG-3' and 5'-AC GAG ACC AAT CAT CAG GAT CTC-3'. The Cyclin D1 primer pairs were 5'-GGA TGC TGG AGG TCT GCG AGG AAC-3' and 5'-GAG AGG AAG CGT GTG AGG CGG TAG-3'. The E2F1 primer pairs were 5'-GAC TGT GAC TTT GGG ACC-3' and 5'-TC TTC ACC TTC ATT CCC-3'. The TERT primer pairs were 5'-TGT TCC TGT TCT GGC TAA TG-3' and 5'-T GCT TGA CCT CCT CTT GTG-3'. C-Myc primer pairs were 5'-TCC CGC GAC GAT GCC CCT CAA CGT TAG CTT CA-3' and 5'-CAC AAG AGT TCC GTA GCT GTT CAA GTT TGT G-3'. The β-actin levels were used as normalized control, and the primers used were 5'-TCA CCC ACA CTG TGC CCC ATC TAC GA-3' and 5'-CAG CGG AAC CGC TCA TTG CCA ATG G-3'.
