# Supplementary material for: The Wedelolactone Derivative Inhibits Estrogen Receptor-Mediated Breast, Endometrial, and Ovarian Cancer Cells Growth
Source: Biomed Res Int. 2014 Aug 13;2014:713263. doi: 10.1155/2014/713263 (PMC4157183; doi:10.1155/2014/713263)
Supplement: Supplementary file 1 — The supplementary document includes detailed methods for I. Cell Culture, II. In Vitro Cell Growth Assay, III. Luciferase Assay, and IV. RNA Extraction, Reverse transcription, and Real-time PCR. [file 713263.f1.zip › 713263.f1/table1.pdf]

Cell	IC50 (μM) (10nM E2)	IC50 (μM)
MCF-7	18.3±2.0	29.6±2.0
Ishikawa	32.2±2.0	63.2±3.5
SKOV-3	41.9±2.5	79.6±4.0
MBA-MD-231	42.5±3.5	43.8±2.5
HEC-1-A	80.4±4.5	84.3±4.5
OVCA429	77.5±4.5	80.3±4.5
